# Investigation of the Quench Sensitivity of an AlSi10Mg Alloy in Permanent Mold and High-Pressure Vacuum Die Castings

**DOI:** 10.3390/ma12111876

**Published:** 2019-06-11

**Authors:** Mengyun Liu, Zhan Zhang, Francis Breton, X.-Grant Chen

**Affiliations:** 1 Department of applied sciences, University of Quebec at Chicoutimi, Saguenay, QC G7H 2B1, Canada; mengyun.liu1@uqac.ca (M.L.); zhan_zhang@uqac.ca (Z.Z.); 2 Arvida Research and Development Centre, Rio Tinto Aluminum, Saguenay, QC G7S 4K8, Canada; francis.breton@riotinto.com

**Keywords:** AlSi10Mg alloy, quench sensitivity, permanent mold casting, high pressure vacuum die casting, heat treatment, microstructure

## Abstract

The quench sensitivities of an AlSi10Mg alloy in permanent mold (PM) and high-pressure vacuum die (HPVD) castings were investigated with time–temperature–transformation and time–temperature–property diagrams using an interrupted quench technique. The quench-sensitive temperature range of the HPVD casting sample is 275–450 °C, and its nose temperature is 375 °C. The quench-sensitive range of the PM casting sample is 255–430 °C, and the nose temperature is 350 °C. The mechanical strength versus the cooling rate in both casting samples were predicted via a quench factor analysis and verified experimentally. The critical cooling rate of the HPVD casting sample is 20 °C/s whereas it is 17 °C/s for the PM casting sample. With a shorter critical time, higher nose temperature, and higher critical cooling rate, the HPVD casting sample exhibits a higher quench sensitivity than the PM casting sample. The differences in the quench sensitivities of the AlSi10Mg alloy due to the different casting processes is explained via the different precipitation behavior. At the nose temperature, coarse β-Mg_2_Si precipitates mainly precipitate along the grain boundaries in the HPVD casting sample, whereas rod-like β-Mg_2_Si precipitates distribute in the aluminum matrix in the PM casting.

## 1. Introduction

Al–Si foundry alloys possess excellent castability, high strength-to-weight ratio, and good corrosion resistance [1]. As one variant in the Al–Si foundry alloy family, the AlSi10Mg alloy (Aural^TM-3^ [1]) is particularly suitable for casting thin-wall and large structural components for a variety of applications in the automotive and aerospace industries [1,2]. The AlSi10Mg cast components are often fabricated via high-pressure die casting and permanent mold (PM) casting. The casting process is one of the key factors affecting the microstructure and mechanical properties. In PM casting, the molten metal is poured at a low speed into a metallic mold under gravity (<1.5 m/s in the gate for aluminum alloys) and solidified under a relatively low cooling rate (<10 KS^−1^ for aluminum alloys) [3,4]. In general, PM castings have uniform microstructures, and their mechanical properties can be significantly improved via heat treatments. By contrast, high pressure die castings require a high melt injection speed (in the range of 40 m/s in the gate for aluminum alloys) and solidify under high pressures (up to 200 MPa in the cavity) with a high cooling rate (up to 10^3^ KS^−1^) [5,6,7], which results in a much finer microstructure but more gas porosity than PM castings. In the high pressure die casting, entrapped air in the cavity can be efficiently evacuated by employing a vacuum technique, which greatly reduce the gas porosity in the cast part. High pressure vacuum die (HPVD) casting makes the cast components heat-treatable.

To obtain heat-treatable AlSi10Mg alloys with high mechanical properties, precipitation-hardening heat treatments (T6 and T7) are generally used [1]. They involve three main steps: solution treatment, quenching, and artificial aging. Quenching is a critical step; inadequate quenching often weakens the mechanical properties during artificial aging. By contrast, excessive rapid quenching can produce a severe residual stress and therefore distortions in the final product. It is crucial to gain sufficient knowledge on the quench sensitivity of heat-treatable alloys under different cast conditions. Time–temperature–property (TTP) and time–temperature–transformation (TTT) diagrams are ideal tools for assessing the quench sensitivity because they deliver important information, such as the critical time and nose temperature for precipitation during quenching [8,9,10]. An interrupted quenching technique with series of isothermal heating tests has been widely used to determine TTP diagrams [11]. Combined with TTP curves, the quench factor analysis introduced by Evancho and Staley in the 1970s [11] is an effective method to establish a relationship between the cooling rate in the quench process and the mechanical properties. 

The quench sensitivity was initially studied in wrought aluminum alloys and later in foundry alloys. Several studies on the quench sensitivity of Al–Si–Mg foundry alloys using different methods have been reported [12,13,14,15,16]. Zhang and Zheng [13] investigated the quench sensitivity of the AlSi7Mg0.4 alloy and discovered that the heat treatment process affected the quench sensitivity. Milkereit et al. [14] studied the precipitation behavior of the AlSi7Mg0.3 alloy during quenching, using a continuous cooling precipitation diagram to obtain the critical cooling rate. Tiryakioğlu and Shuey [12] constructed TTP curves based on the yield strength for the D357 alloy (AlSi7Mg0.62) and reported that this foundry alloy had a slightly lower quench sensitivity than the 6061 alloy (Al–0.65Si–0.89Mg) but exhibited a higher quench sensitivity than the 6082 alloy (Al–0.92Si–0.59Mg), owing to the different solute concentrations. Okayasu et al. [17] studied the mechanical properties of an Al–Si–Cu alloy produced via various casting processes. They reported that the high pressure die casting had much finer α-Al grain and Al–Si eutectic structures than the PM casting. In our previous work [18], the microstructure and mechanical properties of AlSi10Mg permanent mold and high-pressure vacuum die castings were investigated. It was found that the size of the microstructural phases in the HPVD castings was much finer than those in PM castings and there was macro-segregation of Si and Mg in the HPVD castings. Moreover, the evolution of the mechanical properties of HPVD castings on T6 heat treatment is not same as that of PM castings. Because different casting processes result in very different microstructures, the impact of the microstructure on the precipitation behavior during quenching, and subsequently on the quench sensitivity, cannot be neglected. However, very few researchers have studied the impact of the casting process and its related microstructure on the quench sensitivity.

In the present study, the quench sensitivities of an AlSi10Mg alloy in PM and HPVD castings were investigated. The TTT and TTP diagrams were constructed via an interrupted-quench technique. The temperature ranges of the quench sensitivity and the key constants for both casting processes were determined. The influence of the quench rates on the mechanical properties was determined via a quench factor analysis. Finally, the differences in the quench sensitivities were explained based on the different precipitation behaviors.

## 2. Experimental 

The HPVD casting samples were produced with a cold-chamber vacuum die casting machine (Buhler 26D, Markham, ON, Canada) equipped with a Castool vacuum system (Uxbridge, ON, Canada). The dimensions of the cast plates were 220 mm × 65 mm × 2.5 mm. For the PM casting samples, the AlSi10Mg alloy was prepared with commercially pure Al (99.7%), pure Mg (99.9%), Al–25%Mn, Al–25%Fe, and Al–50%Si master alloys. Approximately 3 kg of these materials were melted in an electrical resistance furnace (PYRADIA, Saint-Hubert, QC, Canada) for each batch. The melt was kept at 720–750 °C for 30 min, degassed for 15 min, and then poured into a copper mold preheated at 250 °C. The dimensions of the cast plates were 100 mm × 80 mm × 4 mm. Table 1 lists the chemical compositions of the two casting samples analyzed with an optical emission spectrometer (ARL 3460, ThermoFisher, Mississauga, ON, Canada).

All casting samples underwent a solution heat treatment at 500 °C for 3 h. Afterward, the samples were immediately transferred (below 3 s) to a salt bath at 250–450 °C for a certain duration. The salt bath temperature was continuously monitored and maintained at ±1 °C of the targeted temperature. After isothermal holding, the samples were quenched in cold water and artificially aged at 170 °C for 2.5 h. The electrical conductivity (EC) of the as-quenched samples was measured with a Sigmascope SMP10 EC tester (Windsor, CT, USA). The average value of four measurements was taken. Furthermore, Vickers microhardness tests were conducted after artificial aging on the cross sections of the polished samples using a Nextgen NG-1000CCD hardness test machine (Vancouver, BC, Canada) with a load of 100 g and dwell time of 15 s. The average hardness value was taken from ten measurements. To verify the precipitation kinetics, a differential scanning calorimetry (DSC) analysis was performed with a DSC 8000 calorimeter (Perkin-Elmer, Montreal, QC, Canada) at a heating rate of 10 °C/min on the solution-treated samples. 

The microstructures of the samples were examined with an optical microscope with an image analyzer (CLEMEX software PE4-0, Clemex, Longueuil, QC, Canada), a scanning electron microscope (SEM, JSM-6480LV, JEOL, Tokyo, Japan) equipped with an electron backscatter diffraction system (EBSD), and a transmission electron microscope (TEM, JEM-2100, JEOL) operated at 200 kV. The TEM foils were prepared with a twin-jet machine in a solution of 25% nitric acid and 75% methanol at −25 °C. 

To verify the predicted mechanical properties for various quench rates, the solution-treated samples were quenched in different media (still or forced air, fiberglass insulator, oil with different temperatures), followed by an aging treatment at 170 °C for 2.5 h. Finally, the microhardness of the samples was measured to determine the mechanical properties under various quench rates. 

## 3. Results and Discussion

### 3.1. Time–Temperature–Transformation (TTT) Diagrams

**Figure 1:**
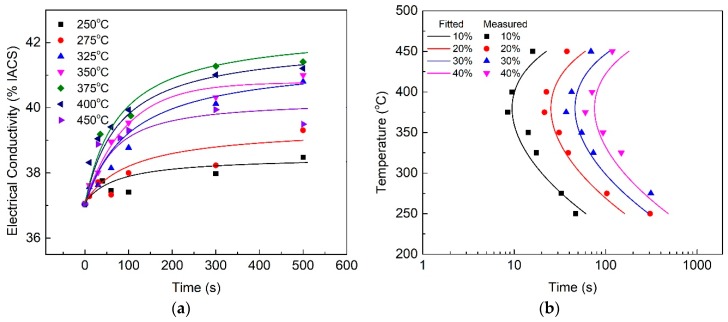
Effect of isothermal treatment on electrical conductivity (EC) of quenched HPVD casting samples (**a**); time–temperature–transformation (TTT) diagram of HPVD casting (**b**). The lines in (**b**) are the fits to the experimental data by using Equation (1).

**Figure 2:**
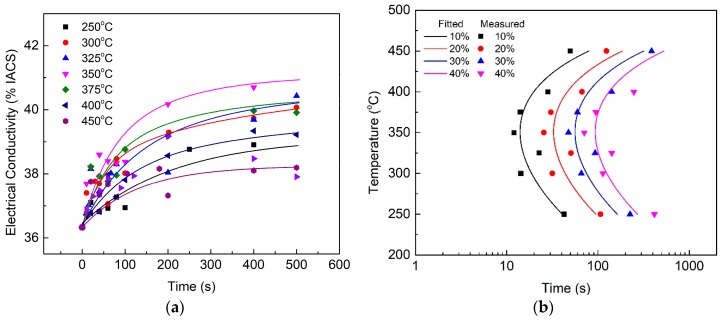
Effect of isothermal treatment on EC of quenched PM casting samples (**a**); TTT diagram of PM casting (**b**). The lines in (**b**) are the fits to the experimental data by using Equation (1).

During isothermal holding, the changes in EC are correlated with the phase transformation, because transforming solute atoms into precipitates will result in an increase in the sample EC. The EC evolutions of the quenched HPVD and PM casting samples after the isothermal treatment are shown in Figure 1a and Figure 2a, respectively. In general, EC increased with an increase in the isothermal holding time. The increase in EC was fast at the beginning of the holding period. Then, the increase rate slowed down until EC reached a stable value. Regarding the isothermal treatment at 375 °C, the growth rate of the EC of the HPVD casting samples was faster than at other temperatures (Figure 1a). The fastest EC growth rate occurred at 350 °C for the PM casting samples (Figure 2a). For the HPVD casting samples, the EC of the solution-treated sample was 37.0%IACS (International Annealed Copper Standard), which represents a supersaturated solid solution state. The EC of the isothermally held sample at 375 °C for 27 h was 42.95%IACS. This represents a complete decomposition of the supersaturated solid solution. For the PM casting samples, the EC of the solution-treated sample was 36.3%IACS, whereas the EC of the isothermally held sample at 350 °C for 27 h was 42.40%IACS. Both EC values of the PM casting samples were slightly lower than those of the HPVD casting samples.

The TTT diagrams were determined via the EC–time curves in Figure 1a and Figure 2a. The measured data and mathematically (Equation (1)) fitted diagrams of the HPVD and PM casting samples are presented in Figure 1b and Figure 2b, respectively. The percentage of the phase transformation in the TTT diagrams corresponds to the percentage of the EC difference between the supersaturated solid solution state and complete decomposition state. Both TTT diagrams possess a “C” shape. The nose temperature of the HPVD casting sample was 375 °C, and the critical time, which represents the incubation of 10% precipitates at the nose temperature, was 10 s. The quench-sensitive temperature range was 275–450 °C. In this temperature range (275–450 °C), EC decreases quickly. Outside the range, EC decreases slowly with an increase in holding time. Thus, the phase transformation rate is high in this temperature range. Regarding PM casting, the nose temperature was 350 °C, which is 25 °C lower than that of the HPVD casting. The quench-sensitive range was 255–430 °C, which is also lower than that of the HPVD casting. The critical time for a precipitate transformation of 10% was 14 s. Hence, the critical time of 10% precipitate transformation for PM casting is 40% longer than that of the HPVD casting. In conclusion, the HPVD casting sample has a shorter critical time, higher nose temperature, and slightly higher quench-sensitive temperature than the PM casting sample.

### 3.2. Time–Temperature–Properties (TTP) Diagrams and Quench Factors

**Figure 3:**
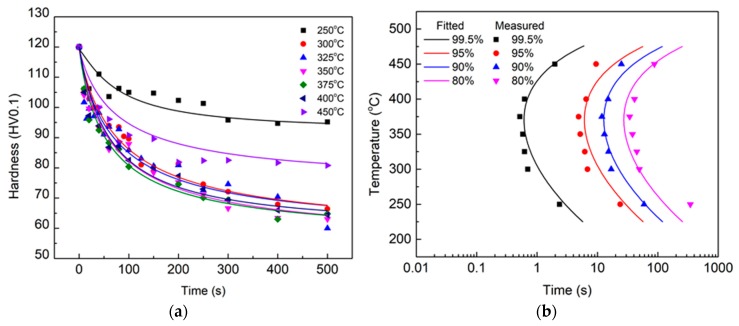
Effect of isothermal treatment on microhardness of quenched HPVD casting samples (**a**); respective time–temperature–property (TTP) diagram (**b**).

**Figure 4:**
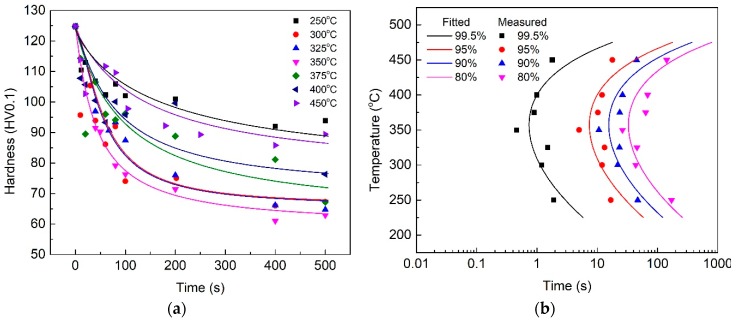
Effect of isothermal treatment on the microhardness of quenched PM casting samples (**a**); respective TTP diagram (**b**).

The mechanical properties of aluminum alloys vary with the precipitation degree during heat treatment. The Vickers hardness of the HPVD casting samples under T6 condition (solution-treated at 500 °C for 3 h; water-quenched and aged at 170 °C for 2.5 h) was 120 HV, whereas it was 125 HV for the PM casting samples. The hardness value under T6 was considered to be the highest hardness of the experimental alloys. Figure 3a and Figure 4a illustrate the hardness evolutions of both HPVD and PM casting samples during the isothermal treatment. In general, the hardness decreased with an increase in isothermal holding time. Similar to the EC curves, the hardness decrease at 375 °C was faster than that at other temperatures for the HPVD casting (Figure 3a). Moreover, the temperature with the fastest hardness decrease was 350 °C for the PM casting (Figure 4a). 

The solute transformation during isothermal holding and the time–temperature–property (TTP) diagram of the precipitation-strengthened alloys can be mathematically described as [11]:

(1) $$C_{t}\left(T\right)=-\;k_{1}k_{2}exp\left[\frac{k_{3}k_{4}^{2}}{RT{\left(k_{4}-T\right)}^{2}}\right]\exp\left(\frac{k_{5}}{RT}\right),$$

where *C_t_*(*T*) is the critical time for a certain amount of solute to precipitate; *k*_1_ is the natural logarithm of the unprecipitated fraction during isothermal holding; *k*_2_ is the constant associated with the reciprocal of the number of nucleation sites; *k*_3_ is the constant corresponding to the energy of a nucleus; *k*_4_ is the constant related to the solvus temperature; *k*_5_ is the constant related to the activation energy for diffusion; R is the gas constant; and *T* is the absolute temperature.

According to Equation (1) and the measured data in Figure 3a and Figure 4a, the TTP diagrams of the HPVD and PM casting samples are shown in Figure 3b and Figure 4b, respectively. The contours of 99.5%, 95%, 90%, and 80% of the maximal hardness are shown in the diagrams. The TTP diagrams also exhibit a “C” shape. The nose temperatures and quench-sensitive temperature ranges in the TTP diagrams of HPVD and PM castings are similar to those in the TTT diagrams. 

**Figure 5:**
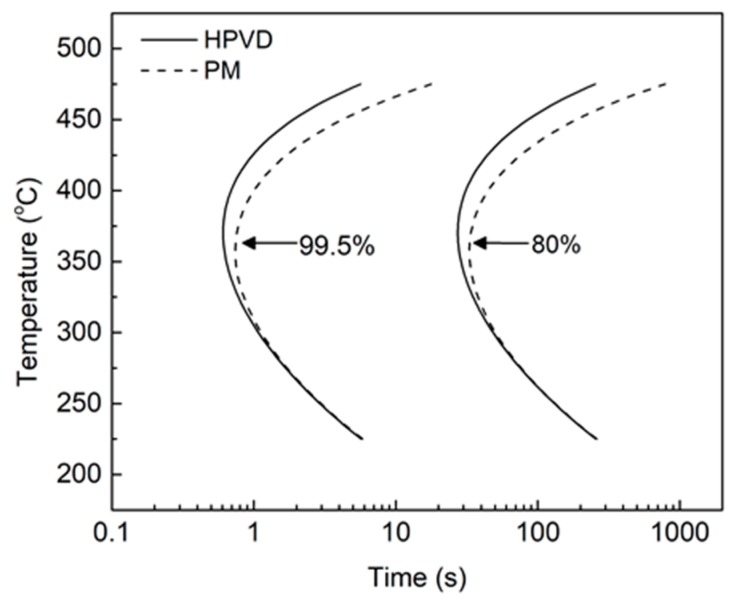
TTP diagrams of 99.5% and 80% of maximal hardness of HPVD (solid) and PM (dashed) castings.

To compare the quench sensitivities of HPVD and PM castings, the TTP diagrams of 99.5% and 80% of the maximal hardness values are illustrated in Figure 5. The time required to obtain 99.5% or 80% of the maximal hardness in the HPVD casting samples was shorter than that for the PM samples when the temperatures is above 300 °C. As the temperature increases, the difference between the required times to obtain 99.5% or 80% maximal hardness increases between the two casting processes. At nose temperature, the critical times for 99.5% and 80% maximal hardness were 0.6 s and 26 s for the HPVD casting, respectively. The corresponding values for the PM casting were 0.8 s and 33 s. Because the HPVD casting samples had a higher nose temperature and shorter critical time than those of the PM casting, it is evident that the precipitation of the former was faster than that of the latter. Hence, the quench sensitivity of the HPVD casting sample is higher than that of the PM casting sample.

The coefficients in Equation (1) were determined by using a non-linear least-squares regression of the measured data. These determined coefficients are shown in Table 2. It can be seen that *k*_2_ of the HPVD casting samples (associated with the reciprocal of the number of nucleation sites) is higher than that of the PM casting samples, which means that the number of active nucleation sites on the HPVD casting samples is less than that in PM casting. The *k*_3_ value of the HPVD casting samples is slightly below that of the PM casting samples, indicating that the energy required to generate nuclei in the HPVD casting samples is less than that of the PM casting samples. Moreover, the *k*_4_ values (related to the solvus temperature) are equal, owing to the similar chemical compositions of both casting samples. The value of *k*_5_ of the HPVD casting is slightly below that of the PM casting samples, thereby indicating that the solutes in the HPVD casting samples are likely to diffuse more readily than those in PM casting samples.

The quench factor, *τ*, can be calculated according to [11]:

(2) $$\tau =\sum_{t_{o}}^{t_{f}}\frac{\Delta t}{C_{t}\left(T\right)},$$

where *C_t_*(*T*) is the critical time necessary for a certain number of solutes to precipitate, as shown in the TTP diagrams; *t_o_* is the time at the start of the quench; and *t_f_* is the time at the end of the quench.

The relationship between the quench rate and mechanical properties was determined based on a quench factor analysis. The predicted strength, σ, can be expressed as [11]:

(3) $$\sigma =\sigma_{max}\exp\left(k_{1}\tau \right),$$

where σ_max_ is the maximal strength attainable with an infinite quench rate.

Next, the decrease of the mechanical properties can be determined via [8]:

(4) $$\Delta \%=1-\exp(k_{1}\tau ),$$

where ∆ is the hardness decrease; *k*_1_ and τ were taken from Equations (2) and (3). 

**Figure 6:**
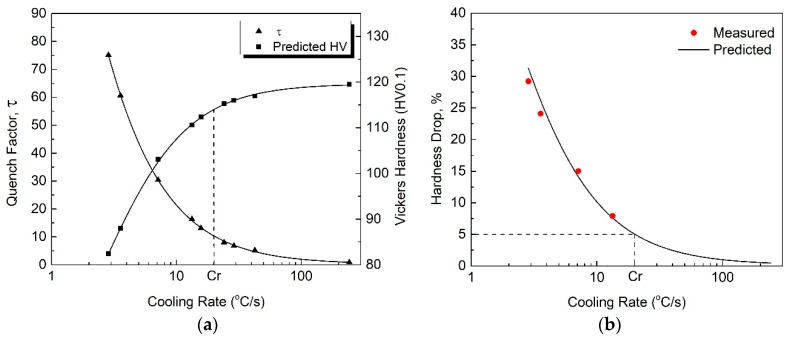
Effect of cooling rate on quench factor and predicted hardness (**a**) and comparison between measured data and predicted hardness decrease (**b**) of HPVD casting samples. *Cr* is the abbreviation of the critical cooling rate.

**Figure 7:**
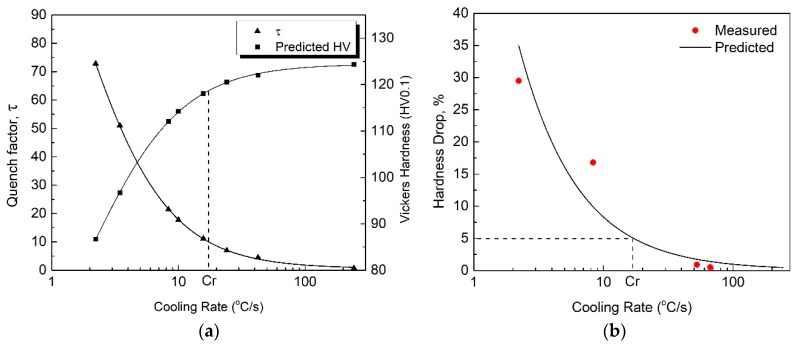
Effect of cooling rate on quench factor and predicted hardness (**a**) and a comparison between the measured data and predicted hardness decrease (**b**) of PM casting samples.

To ensure the accuracy of the calculated results, ∆t = 0.1 s was selected in Equation (2) because the temperature decrease during each time step should be below 25 °C [19]. According to [20], if the quench-sensitive temperature range is considered in the quench factor calculation, the influence of the temperature range on the calculation can be neglected. In the present study, the temperature range 250–450 °C was used for the quench factor calculation in order to cover the quench-sensitive temperature ranges of the HPVD (275–450 °C) and PM casting samples (255–430 °C). The effect of the cooling rate on the quench factor and predicted hardness in the HPVD and PM casting samples is shown in Figure 6a and Figure 7a. It is evident that the quench factor decreases and the predicted hardness increases with an increase in cooling rate. Furthermore, the quench factor decreases rapidly until it reaches a certain cooling rate, *Cr*, where the corresponding hardness is 95% of the maximal value. When the cooling rate is higher than *Cr*, the predicted hardness increases slowly. Regarding the HPVD casting, *Cr* was determined to be 20 °C/s, whereas it was 17 °C/s for the PM casting.

To verify the effect of the cooling rate on the predicted hardness, the solution-treated samples were quenched in different media (still or forced air, fiberglass insulator, oil with different temperatures) with different cooling rates. Afterwards, they were artificially aged at 170 °C for 2.5 h. The hardness of the samples was measured and plotted against the cooling rate. Their values are presented in Figure 6b and Figure 7b, in which the dots are the measured data, and the lines are the predictions based on Equation (4). The predicted hardness agrees well with the experimental results, and the hardness decreases with an increase in cooling rate. When the cooling rate is higher than the critical rate, *Cr* (20 °C/s for HPVD and 17 °C/s for PM), a hardness decrease below 5% (i.e., more than 95% of the maximal value) can be obtained in both casting processes. Moreover, the HPVD casting sample needs a higher cooling rate to obtain the same properties. This is more evidence for the higher quench sensitivity of HPVD casting samples compared with that of PM casting samples. 

### 3.3. Microstructure Observation and DSC Analysis 

**Figure 8:**
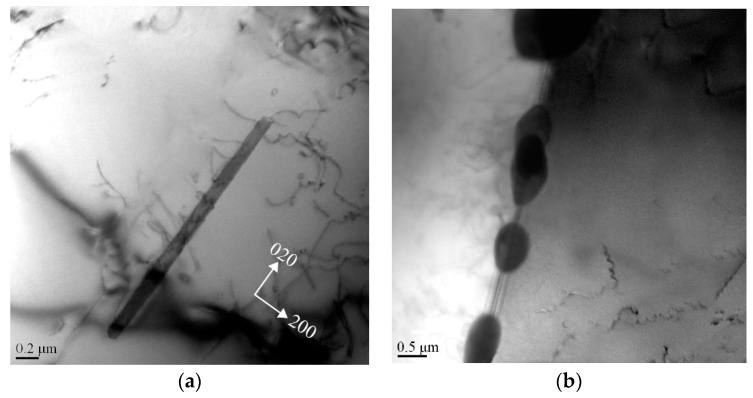
Bright-field transmission electron microscope (TEM) images of precipitates in the aluminum matrix (**a**) and along the grain boundary in the HPVD casting samples (**b**); isothermal holding at nose temperature (375 °C) for 300 s, recorded near [001] direction.

**Figure 9:**
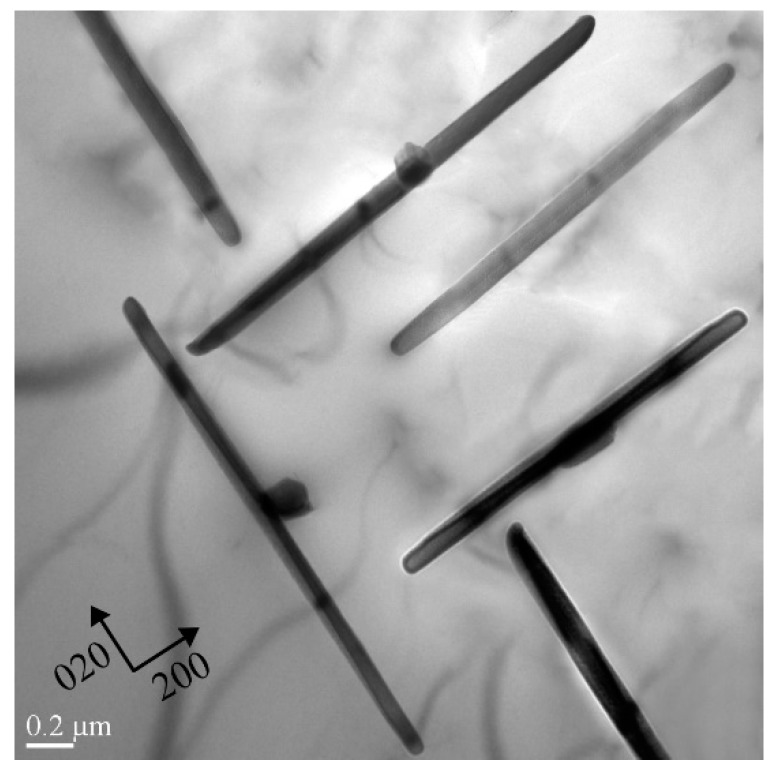
Bright-field TEM image of precipitates in the PM casting samples; isothermal holding at nose temperature (350 °C) for 300 s, recorded near [001] direction.

To investigate the microscopic mechanisms of the quench sensitivity, the heterogeneous precipitation behavior of the HPVD and PM casting samples at the nose temperature was examined. Figure 8 presents the TEM micrographs of the HPVD casting samples after isothermal holding at 375 °C for 300 s. As shown in Figure 8a, only a few rod-like precipitates with lengths of 1–2 µm and thicknesses of 15–25 nm can be observed in the aluminum matrix. However, many granular particles with diameters of 0.5–2 µm precipitated along the grain boundaries (Figure 8b). Those coarse rod-like and granular precipitates were the equilibrium β-Mg_2_Si phase, identified based on the work of reference [21]. Evidently, the grain boundaries act as effective nucleation sites for the coarse equilibrium β-Mg_2_Si phase at the nose temperature, which leads to a high quench sensitivity. Figure 9 shows the TEM micrograph of the PM casting samples after isothermal holding at 350 °C for 300 s. In contrast to the HPVD casting samples, in the PM casting samples, many rod-like β-Mg_2_Si phases with lengths of 1–3 µm and thicknesses of 20–50 nm precipitated in the aluminum grains. Occasionally, a few granular Mg_2_Si particles can be found along the grain boundaries.

**Figure 10:**
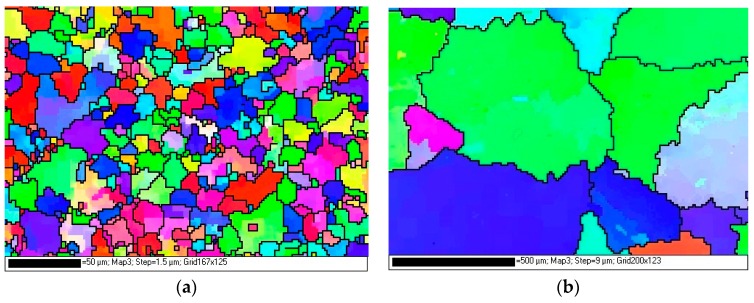
EBSD (electron backscatter diffraction) mappings of solution-treated HPVD casting (**a**) and PM casting samples (**b**). The dark lines indicate the grain boundaries with a misorientation of more than 15°.

Owing to the different solidification conditions of both casting processes, the grain sizes of the HPVD and PM casting samples were different, and they were examined via EBSD. The results are shown in Figure 10. In those EBSD mappings, the black lines indicate the grain boundaries (misorientation of more than 15°). Table 3 lists the statistical data of the grain sizes and lengths of the grain boundaries of the HPVD and PM casting samples. Evidently, the average grain size of the HPVD casting samples was much smaller (approximately 22 times) than that of the PM casting samples. Consequently, the average length of the grain boundaries of the HPVD casting samples was 15 times longer than that of the PM casting samples. Based on the above TEM results (Figure 8 and Figure 9) and owing to the numerous grain boundaries of the HPVD casting samples, it can be concluded that the majority of coarse β-Mg_2_Si precipitated along the grain boundaries in the HPVD casting sample, whereas in the PM casting sample, the coarse β-Mg_2_Si precipitates were mainly distributed within the aluminum grains.

Generally, the quench sensitivity is closely related to the conditions of the phase nucleation and growth near the nose temperature [22]. Owing to abundant defects in the crystal structure, the grain boundaries can provide favorable nucleation sites as well as a fast diffusion for solutes for the subsequent growth. As observed, the HPVD casting samples have many more grain boundaries than the PM casting samples. The precipitation of β-Mg_2_Si in the HPVD casting samples should be easier and faster than in the PM casting samples, thereby resulting in a higher quench sensitivity for the HPVD casting samples.

**Figure 11:**
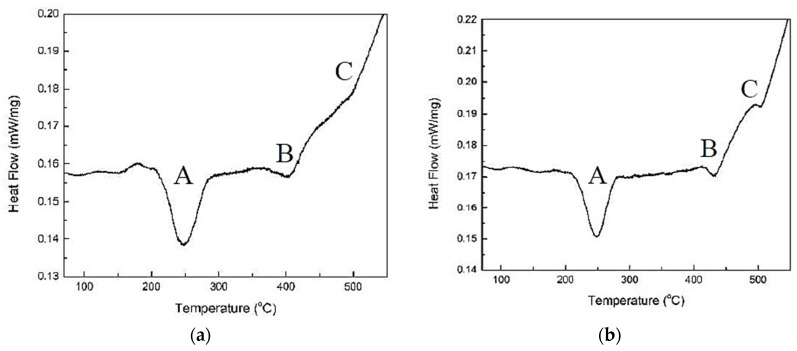
Typical differential scanning calorimetry (DSC) heating curves after solution treatment (500 °C for 3 h) of the HPVD (**a**) and PM (**b**) casting samples.

Furthermore, a non-isothermal DSC analysis was conducted to examine the precipitation kinetics of different Mg_2_Si phases after the solution treatment of the HPVD and PM casting samples. Figure 11 demonstrates typical DSC heating curves of the HPVD and PM casting samples for a heating rate of 10 °C/min. The DSC curves reveal three exothermic peaks: A, B, and C. Table 4 lists the corresponding data related to the peaks. Peaks A, B, and C represent the precipitations of β”-, β’-, and β-Mg_2_Si, according to [23], respectively. The HPVD and PM casting samples had approximately the same onset and peak temperature in peak A (β”-Mg_2_Si). However, the onset and peak temperature of peaks B and C of the HPVD casting samples were lower than those of the PM casting samples. Hence, the precipitation of β’- and β-Mg_2_Si in the HPVD casting samples occurred earlier (at a lower temperature) than those in the PM casting samples. Regarding the equilibrium β-Mg_2_Si that precipitated at the nose temperature, the onset and peak temperatures of peak C of the HPVD casting sample occurred at 453 °C and 498 °C, respectively. They are lower than those of the PM casting sample (485 °C and 502 °C, respectively). This is further evidence that the precipitation of β-Mg_2_Si in HPVD casting samples is easier than in PM casting samples.

Moreover, according to the calculated coefficients of the TTP curves (Table 2), the value of *k*_3_ (corresponding to the energy necessary to form nuclei) and the value of *k*_5_ (related to the activation energy necessary for diffusion) of the HPVD casting samples are lower than those of the PM casting samples. A comparison of the *k_3_* and *k_5_* of HPVD and PM suggests that less energy is required to form a nucleus, as well as faster diffusion for its growth in HPVD casting samples than in PM casting samples. This implies that the nucleation and growth of β-Mg_2_Si precipitates in HPVD castings are faster than in PM castings. Finally, the results agree well with the results of the abovementioned microstructure and DSC analyses. 

## 4. Conclusions

(1) The TTP and TTT diagrams of an AlSi10Mg alloy in PM and HPVD castings were constructed. The sensitive temperature range, nose temperature, coefficients *k*_2_–*k*_5_, and critical cooling rate of both casting samples were determined. According to the results, they were influenced by the casting process.
(2) The quench-sensitive temperature range of the HPVD casting sample was 275–450 °C, and its nose temperature was 375 °C. The quench-sensitive range of the PM casting sample was 255–430 °C, and the nose temperature was 350 °C. The critical time at the nose temperature of the HPVD casting sample was shorter than that of the PM casting. With a shorter critical time and higher nose temperature, the HPVD casting sample possesses a higher quench sensitivity than the PM casting.
(3) The hardness versus the cooling rate in both castings was predicted using quench factor analysis and verified by the experimental results. The critical cooling rate *Cr* of the HPVD casting sample was determined to be 20 °C/s, whereas it was 17 °C/s for the PM casting sample. When the cooling rate is higher than *Cr* during quenching, more than 95% of the maximal mechanical strength can be obtained in both casting processes.
(4) The difference in the quench sensitivities of the AlSi10Mg alloy in the two casting processes is mainly due to the different precipitation behaviors. At the nose temperature, coarse β-Mg2Si precipitates prefer to nucleate and grow along the grain boundaries in HPVD casting samples, whereas rod-like β-Mg2Si precipitates distribute in the aluminum matrix in PM casting samples.

## Figures and Tables

**Table 1:** Chemical composition (wt%) of high pressure vacuum die and permanent mold castings.

Castings	Al	Si	Fe	Mn	Mg	Ti	Sr
HPVD	bal.	10.1	0.18	0.50	0.55	0.06	0.012
PM	bal.	10.1	0.20	0.50	0.59	0.06	0.011

**Table 2:** Coefficients for TTP diagram of HPVD and PM.

Castings	*k*_2_ (s)	*k*_3_ (J mo^−1^)	*k*_4_ (K)	*k*_5_ (J mol^−1^)
PM	0.35 × 10^−6^	3103	955	77243
HPVD	1.85 × 10^−6^	2483	955	72966

**Table 3:** Grain size and grain boundary length of HPVD and PM castings.

Castings	Equivalent Grain Diameter (µm)		Grain Boundary Length (µm/µm^2^)	
	Average	Standard Deviation	Average	Standard Deviation
HPVD	4.4	3.5	11.28	0.71
PM	95.2	40.2	0.67	0.18

**Table 4:** Average values of onset and peak temperatures of precipitation in HPVD and PM castings.

Castings	Peak A		Peak B		Peak C	
	Onset T (℃)	Peak T (℃)	Onset T (℃)	Peak T (℃)	Onset T (℃)	Peak T (℃)
HPVD	214	243.50	360	405	453	498
PM	214	243.10	413	434	485	502
